# **^s^** CIRCLE—An interactive visual exploration tool for single cell RNA-Seq data

**DOI:** 10.1093/nargab/lqae084

**Published:** 2024-07-17

**Authors:** Maximilian Seeger, Erich Schöls, Lars Barquist

**Affiliations:** Technical University of Applied Sciences Würzburg-Schweinfurt, Faculty of Design, 97070 Würzburg, Germany; Helmholtz Institute for RNA-based Infection Research, Helmholtz Centre for Infection Research, 97080 Würzburg, Germany; Technical University of Applied Sciences Würzburg-Schweinfurt, Faculty of Design, 97070 Würzburg, Germany; Helmholtz Institute for RNA-based Infection Research, Helmholtz Centre for Infection Research, 97080 Würzburg, Germany; University of Würzburg, Faculty of Medicine, 97080 Würzburg, Germany; Department of Biology, University of Toronto Mississauga, Mississauga, ON, L5L 1C6, Canada

## Abstract

^s^CIRCLE (**s**ingle-**C**ell **I**nteractive **R**eal-time **C**omputer visualization for **L**ow-dimensional **E**xploration) is a tool for exploratory analysis of single cell RNA-seq (scRNA-seq) data sets, with a focus on bacterial scRNA-seq. The software takes an information design perspective to re-envision visually and interactively exploring low dimensional representations of scRNA-Seq data. Users can project cells in various 3D and 2D spaces and interactively query and paint cells using rich metadata sets reporting on cell cluster, gene function, and gene expression. As a standalone application it contains, among other features, options for dimensionality reduction, navigation and interaction with data in 3d and 2d space, gene filtering, fold change and metacell computation as well as various capabilities for visualization, data import and export.

## Introduction

The rapid development of single-cell omics techniques and the growing size of single cell RNA-Seq (scRNA-seq) data sets not only presents new opportunities for unraveling biological mechanisms related to heterogeneity within cell populations, but also poses major challenges for analysis and visualization of these data sets (1). A growing number of emerging tools in this field focussing on visualization, dimensionality reduction and clustering (2), show that there is a perceived need for new approaches to visualizing and exploring high-dimensional scRNA-Seq data sets. A comparison of several existing visualization tools (3) calls on designers and developers alike to not only focus on efficiency and scalability, but also to invent new features to enrich data visualization and stand out from existing tools. The authors also emphasize the need of visual tools to enable communication about data sets with collaborators and colleagues using quick and easy data exploration features.

sCircle was developed through a close collaboration between designers and computational biologists. It has previously been noted that there is great potential for collaboration between bioinformaticians and designers, but there is a lack of natural intersections between the two communities (4). In this project, we embedded a design student within a computational biology research group who took a research through design (5) approach to building a scRNA-seq visualization tool. This involved regular consultations with experimental and computational practitioners throughout the design process with the goal of developing new visual languages to make visual problem solving (6) easier, faster, and more accurate for experts in the field.

^s^Circle offers new interactive visual approaches to explore and mine single cell-level gene expression data, enriched with functional annotation data to facilitate discovery in scRNA-Seq data sets focused on bacterial scRNA-seq (7). Of the criteria previously evaluated for single-cell visualization tools (3) ^S^Circle includes easy cell selection, zooming and navigation options, multiple embedding options, the possibility to highlight expression of particular genes, and enhanced metadata and cell-type annotations. The software offers extensive possibilities for gene filtering based on user-supplied metadata and the ability to compute metacells, that is, averaged expression profiles for defined groups of cells. Currently ^S^Circle offers calculation of fold-changes between cells and metacells as a heuristic means for differential expression analysis. A video tutorial showcasing the features of ^S^Circle is available at https://www.youtube.com/watch?v=o5RBYT8c8E0.

## Materials and methods

### Implementation

This tool was implemented using vvvv, a C#-based visual programming language and the source code is publicly available. The freely available vvvv editor is needed to view and edit the source code. The Accord.NET framework was used for the implementation of basic statistical functions. For constructing the user interface, the vvvv-specific Elementa package was used. Performance was optimized for larger data sets and use with consumer hardware by limiting calculations performed at runtime. The C# observer design pattern was used internally to compute functions like filters, fold changes, metacells or annotation data queries in parallel with the main application, notifying and updating the main process only when calculations have finished in the background. Performance-heavy calculations are outsourced to a different thread. To enable the efficient rendering of many cells, instanced drawing has been used to render bars and lines, while sphere imposters (8) have been implemented for efficient rendering of spheres. On a 2016 consumer laptop (i5, onboard graphics card, 8GB RAM) benchmarking demonstrated usability for data sets up to 15.000 cells (1000 cells, 90 fps; 5000 cells, 30 fps; 15 000 cells, 15 fps). On a desktop PC (i7, Nvidia GTX 1080TI, 32 GB RAM) the software still maintained usable frame rates with over 50 000 cells (5000 cells, 90 fps; 15 000 cells, 80 fps; 50 000 cells, 30 fps).

All filters have been implemented as delegates so that they can be invoked in any order and the same filter function can be called multiple times in a row. This also allows for an easy implementation of additional filters.

The final software was built as an executable file only dependent on the installation of the Microsoft.net 5.0 framework. sCircle is publicly available alongside an example data set (9) and in-depth documentation at https://github.com/BarquistLab/sCIRCLE. The software has been tested on Windows 10 and 11 and can also be run in a virtual machine on OS X using Parallels or BootCamp. As the rendering pipeline only offers experimental support for Virtual Machines on Linux systems, sCircle is not officially supported on Linux Virtual Machines.

### Data import

Data sets can be imported using three separate files containing a count matrix, gene annotations, and phenotypic data following specified formatting and naming conventions. We note that our software assumes data has undergone appropriate quality control measures, for instance to filter cell doublets or dead cells. Supported file formats are CSV, Excel or GFF. Example data files are available at https://github.com/BarquistLab/sCIRCLE/tree/main/Example_DataSet. The count matrix must include a first column containing a unique identifier for each cell, with the first row containing unique identifiers for each gene. While sCircle is designed to display scRNA-Seq data sets, any properly formatted matrix including counts or numeric values could be imported. The second file contains gene annotation data. The first column must include identifiers matching those in the count matrix. Every additional column in the gene annotation file can include a category for metadata for each gene, while the first row specifies the name of the category. For example, one column could contain GO-terms per gene, while another column could contain associated KEGG Pathways. Additionally, gene groups can be defined by starting a column name with ‘G_’ and adding a ‘1’in the table for each gene, which should be part of the group. This can be used to import known groups of associated genes like pathogenicity islands or to accelerate filtering for genes of interest in certain experimental scenarios. There is no minimum or maximum requirement for the number of gene annotation categories, but the file must at least include one row with locus tags for the given genes. Gene annotation files from third-party tools like eggNOG-mapper (9) can be imported with minimal formatting changes.

Similarly to the gene annotation file, the phenotypic data file can include metadata per cell or experimental condition of the imported data set. This could be used, for example, to label cells from different experimental conditions or associate them with different batches. This file can also be used to import pre-computed clusters. The first column of the file must include the unique identifiers for each cell matching identifiers from the first row of the count matrix. Every other column can include a category, which can be used to color cells accordingly inside the software or to calculate average gene expression across all genes for all cells inside a cluster.

The workflow of the software is designed in a way that all three tables can be easily manipulated in e.g. R or Python for more extensive data analysis or quality control, then exported to go back and forth between data analysis and exploratory visualization. There are some naming conventions that must be followed to allow correct data parsing. Custom sets of genes, which can be used as a filter inside the software, can be defined within the gene annotation file. The column containing the Locus Tags must be called ‘Locus_Tag’ and the columns containing start and end points of each gene must be called ‘Start’ and ‘End’ (case sensitive). Multiple entries in one cell of a table must be separated with a comma. Gene groups can be imported with the annotation file to be used as a filter inside the software.

## Results

### Functionalities

^S^Circle features an extensive tool set offering possibilities to filter for defined pathways and gene sets, query and mine gene annotation data, compute metacells, compare cells and metacells with fold-changes, annotate and export custom clusters, save genes of interest and interactively navigate PCA or UMAP plots both in 3D and 2D—all in real-time. Functional gene annotation data can be provided by the users following the formatting criteria documented on the project page (https://github.com/BarquistLab/sCIRCLE/). The included example data set uses bacterial gene annotations from eggNOG-mapper (9), including GO terms, KEGG pathways, and COG categories. When starting a new session, a custom data set can be loaded using three separate files—a raw or normalized count matrix, a gene annotation file and a phenotypic annotation file (see Materials and Methods). To reduce the dimensionality of an imported data set, the user can choose between three options: PCA, UMAP or an imported precomputed matrix defining a cell coordinate system. While the PCA implementation is using the Accord.NET framework, the UMAP implementation is based on the UMAP Sharp.net NuGet. Alternatively, users can also supply their own precomputed coordinate matrix that must have at least two dimensions and the number of cells must correspond with the imported count matrix. When using PCA, the user can dynamically swap axes for different principal components and adjust the explained percentage of the variance or the number of calculated principal components. For calculating UMAP the user can switch between a 2D and 3D version as well as adjust the number of neighbors considered.

**Figure 1.**
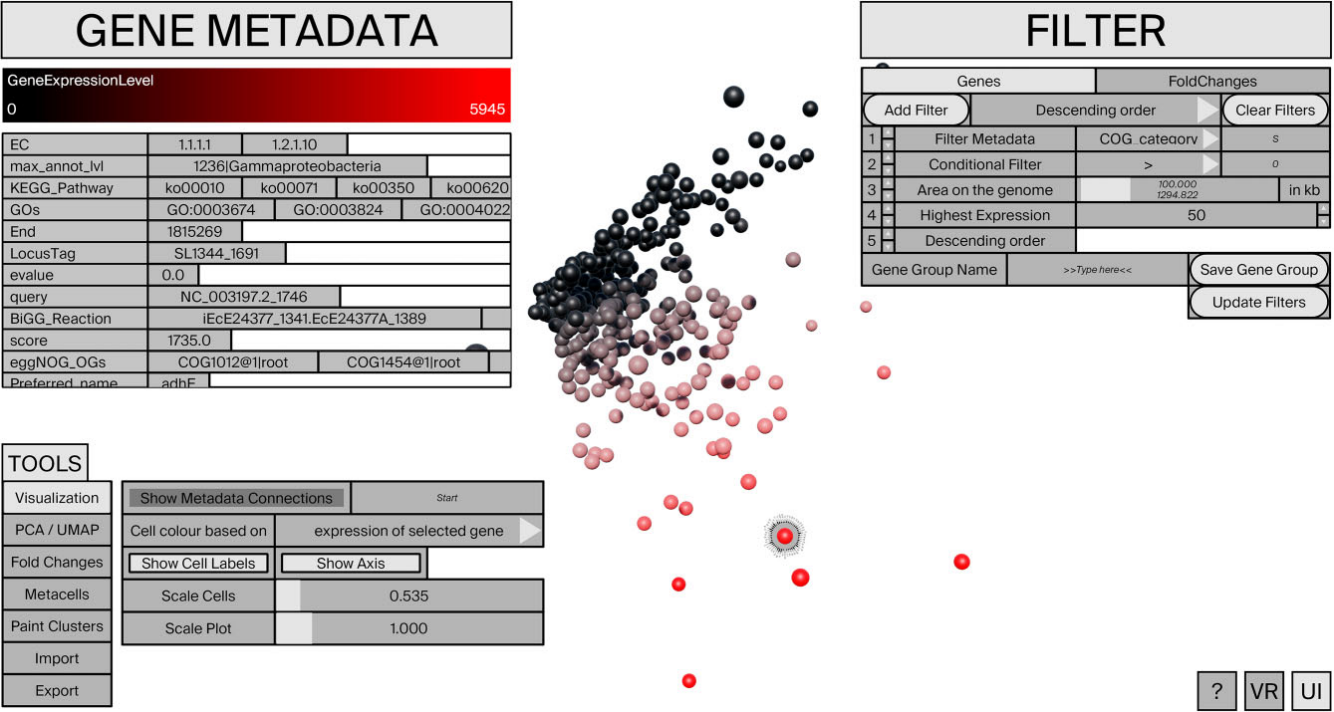
Overview of the application, with all menus open and a 3D PCA plot displayed, using a bacterial scRNA-seq dataset (Homberger *et al.*, 2023). Shades of red display the gene expression for a selected gene across all cells.

The software includes a modular filter set to identify genes and cells of interest (Figure 1). This includes filtering for sets of genes with the highest (differential) expression, those falling within a specific region of the genome, or filtering based on metadata annotations e.g. membership in a certain KEGG Pathway. The user can also filter for custom gene sets. The filter system is built with a modular approach so that all filters are chainable in any order.

**Figure 2.**
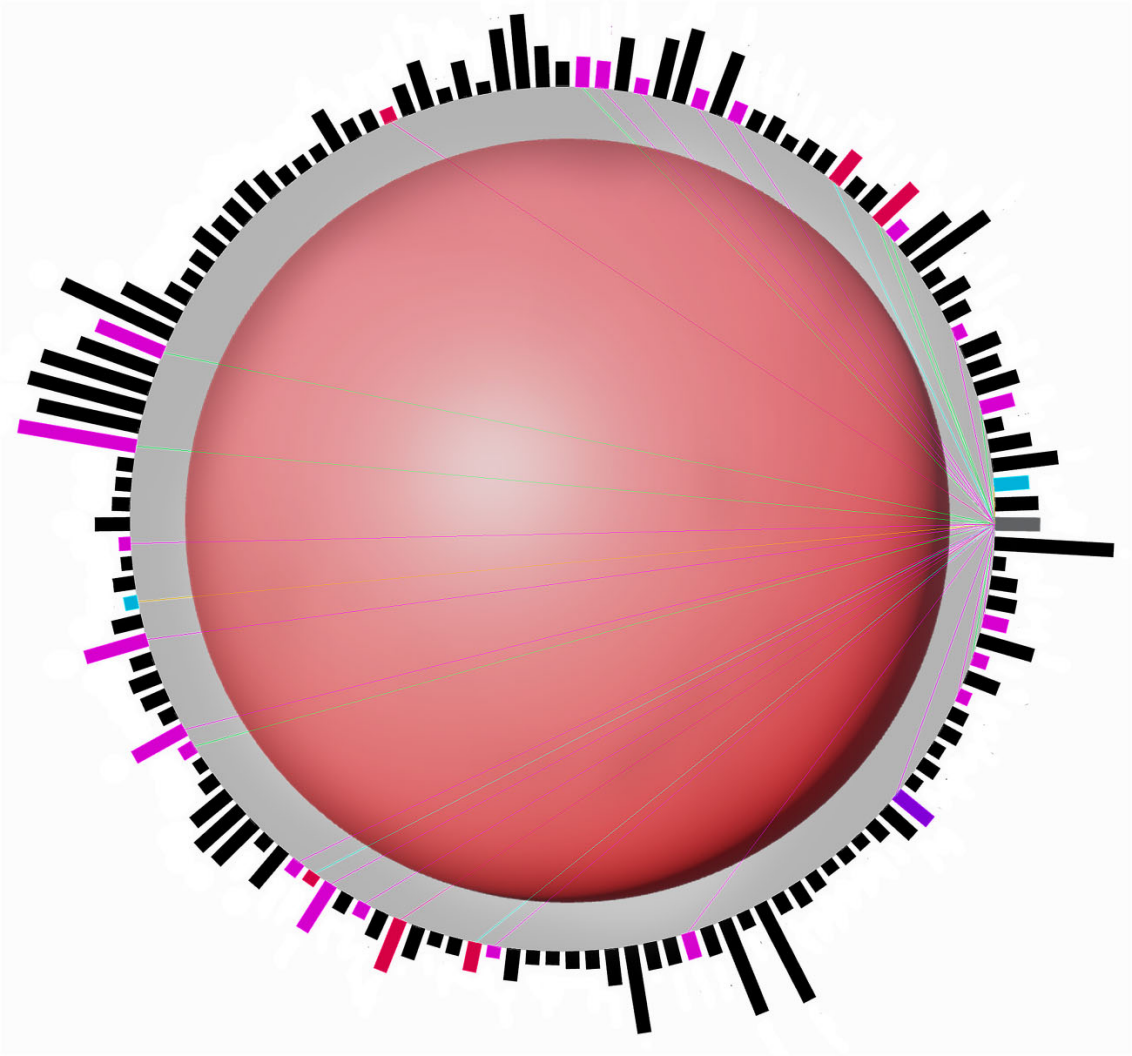
Interactive radial gene expression diagram for a selected cell shows expression levels of the top 50 most highly expressed genes in the cell. Bar heights indicate expression levels (e.g. read counts). The selected gene is displayed in gray, and other genes in the same KEGG pathway as the selected gene are color-coded by pathway membership and linked by colored lines.

The core visualization concept of ^s^Circle is based on nesting interactive radial gene expression charts within an interactive PCA or UMAP scatter plot. Each plot can be freely scaled and rotated in three dimensions. Selecting any cell inside the scatter plot displays the gene expression chart of the selected cell with all currently applied gene filters in real-time (see Figure 2). Each gene within the radial bar plot is clickable and provides access to all annotation data, displaying it in an instant in the metadata inspector in the upper left corner (see Figure 1, ‘Gene Metadata’). Genes can additionally be colored by metadata. For instance, when selecting KEGG Pathways all genes that share a pathway annotation with the currently selected gene appear colored, with each color depicting a different pathway (Figure 2). The colors of the cells can be changed based on imported cluster definitions, for example, different time points or batches or custom coloring options. Alternatively, all cells can be coloured according to the gene expression of a selected gene in each cell adding a heat map functionality to the 3D scatter plot (see Figure 1). Differential gene expression between two cells can be visualized by calculating log fold-changes in real-time and displayed as bar charts. When activated, the log_2_ fold change between the last two selected cells is continuously calculated in the background. For filtering genes displayed in the log fold-change graph, a separate filter menu is available.

The software also features a simple approach to computing metacells based on predefined or user painted clusters of cells or experimental conditions imported with the phenotypic data file. This is especially useful for data sets containing a large number of cells and to identify genes that strongly differ in their expression between different clusters in exploratory analyses. The user can choose to calculate the mean or median gene expression for each gene over all cells belonging to a cluster or condition, for example a specific time point or growth condition. It is important to note that currently the user can not remove outliers from predefined clusters within ^S^Circle, but this can be overcome by manually defining clusters using the paint clusters tool or editing the source cluster file. When the metacell feature is active, every cluster is represented by a single sphere in the scatter plot—a metacell—which can be treated as any other cell inside the software to filter the gene expression charts. The user can also calculate log fold-changes between metacells, calculated as the log_2_ fold-change in mean or median expression between clusters.

All radial expression charts or logFoldChange graphs can be exported as SVG or PNG files. Additionally a dimensionality reduced matrix can be exported as a CSV file either for further analysis with a different software or for saving calculation time, when visualizing the same data set in a new session. Moreover, the gene annotation file can be exported with additional columns for each gene group annotated in the software, and the phenotypic annotation file can be exported with additional columns for every saved cell group. The application also features an experimental VR mode, which can be used to navigate 3D scatter plots with VR glasses using OpenVR.

### sCircle visualizes dynamic expression over growth in bacterial scRNA-seq

**Figure 3.**
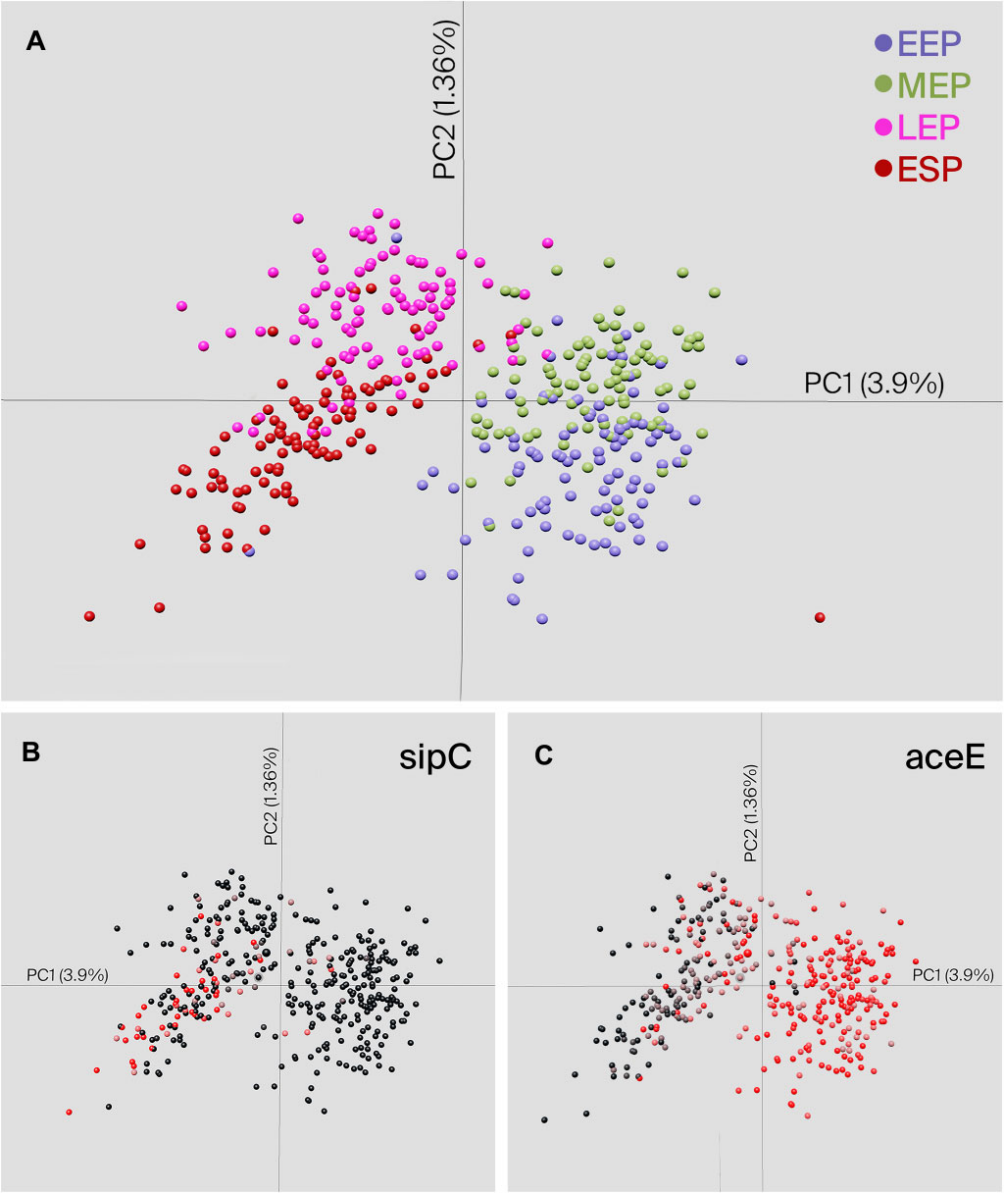
(**A**) PCA plot for single *Salmonella* cells harvested from early exponential phase (EEP), mid-exponential phase (MEP), late exponential phase (LEP) or early stationary phase (ESP) cultures, colored by growth phase. (**B**) Gene expression heatmap in single cells for *sipC*, colored from no expression (black) to high expression (red). (**C**) Gene expression heatmap for *aceE*, colored as in B.

To demonstrate the utility of ^s^Circle, we have reproduced some findings of a recent bacterial scRNA-seq study profiling *Salmonella* Typhimurium gene expression during growth in lysogeny broth (10). Even without extensive normalization and quality control the PCA analysis shows a clear clustering of the cells by time-point, particularly illustrating the differences in *Salmonella* gene expression between exponential growth and stationary phase (Figure 3A). Furthermore, using ^s^Circle's heatmap capabilities we could easily reproduce plots illustrating differential gene expression across growth phases. For instance, the gene encoding the host cell invasion-associated type 3 secretion system (T3SS) effector SipC, responsible for inhibiting phagosome-lysosome fusion, can be clearly seen to be highly expressed in late exponential and early stationary phase (Figure 3B) in agreement with known *Salmonella* expression patterns (11). Only a subset of cells is observed to express *sipC*, recapitulating known heterogeneity in virulence gene expression by *Salmonella* (12). Similarly, *aceE*, encoding a component of the pyruvate dehydrogenase complex responsible for catalyzing conversion of pyruvate to acetyl-CoA, is highly expressed in early and mid-exponential phase (Figure 3C) illustrating the metabolic activity of rapidly dividing cells. Additional features like the comparison of average gene expression between the clusters with metacells or 3D PCA could be used at this stage to visually explore the data set further.

## Discussion

In summary, ^S^Circle is a new tool for scRNA-Seq data sets with a focus on real-time data visualization and interactive exploration, while interlacing gene expression and functional annotation data. While the software already features an extensive tool set for visualization and data exploration purposes, additional statistical features like sophisticated options for normalization and drop-out correction as well as farther reaching methods for differential expression analysis could further extend the applications use cases from a data exploration tool to a visual data analysis pipeline. A current exciting approach to dimensionality reduction uses variational autoencoders (13), promising a more meaningful low-dimensional representation of scRNA-Seq data sets. ^S^Circle provides a powerful interface for exploring the meaning of the latent spaces produced by such techniques. Additionally, virtual spaces promise new approaches to investigating the finer details of high-dimensional data sets using VR or AR technology. While this application currently only offers basic VR integration, creating easy to use virtual spaces for data exploration and designing novel features will be a major project for future work.

In sum, we believe that ^S^Circle offers a new interactive visual approach to exploring scRNA-Seq data sets compared to existing tools. ^s^Circle will be especially useful for biologists without an extensive bioinformatic skill set, as an explorative entry point to data analysis for data scientists, and as a communication tool when collaboratively exploring a data set or communicating findings.

## Data Availability

Source code and documentation are freely available under an MIT license on GitHub. The application is implemented using the C#-based visual programming language vvvv. The available software is compiled as an executable file and can be easily installed on any Windows 10 or 11 system without dependencies. An example data set as well as an in-depth documentation is publicly available on GitHub (https://github.com/barquistlab/scircle) and Zenodo (https://doi.org/10.5281/zenodo.12636724). It is recommended to use the software with a 3-button mouse and a keyboard. A video trailer demonstrating key features is available at https://www.youtube.com/watch?v=eoBJmHXsp9w, and a full video tutorial at https://www.youtube.com/watch?v=o5RBYT8c8E0.
