# Correction to ‘^s^CIRCLE—An interactive visual exploration tool for single cell RNA-Seq data’

**DOI:** 10.1093/nargab/lqae107

**Published:** 2024-08-09

This is a correction to: Maximilian Seeger, Erich Schöls, Lars Barquist, ^s^CIRCLE—An interactive visual exploration tool for single cell RNA-Seq data, *NAR Genomics and Bioinformatics*, Volume 6, Issue 3, September 2024, lqae084, https://doi.org/10.1093/nargab/lqae084

The first author's name has been corrected.

